# Effect of Flat Cornea on Visual Outcome after LASIK

**DOI:** 10.1155/2015/794854

**Published:** 2015-11-29

**Authors:** Engy Mohamed Mostafa

**Affiliations:** Ophthalmology Department, Sohag University, Naser City, Sohag 82524, Egypt

## Abstract

*Purpose*. To evaluate the effect of preoperative and postoperative keratometry on the refractive outcome after laser in situ keratomileusis (LASIK) for moderate and high myopia.* Methods*. Records of 812 eyes (420 patients) with myopia ≥−6 D who had LASIK at Sohag Laser Center, Egypt, from January 2010 to November 2013, were retrospectively analyzed. Main outcome measures were postoperative corrected distance visual acuity (CDVA), postoperative spherical equivalence, and postoperative *Q* factor.* Results*. LASIK was performed in 812 eyes (mean age 21.8 ± 5.2 years). Patients were grouped according to the degree of preoperative myopia into three groups: Group 1, −6 D to −7.9 D; Group 2, −8 to −9.9 D; and Group 3, −10 to −12 D. The refractive outcome among the different myopia groups was stratified by pre- and postoperative keratometry. A trend toward greater undercorrection was noted in eyes with preoperative keratometry <43.5 D compared with those with steeper keratometry >46 D in all myopia groups. The undercorrection was also noted in postoperative keratometry groups <35 D.* Conclusions*. Preoperative and postoperative keratometry appeared to influence the refractive outcome especially in high myopic eyes.

## 1. Introduction

The principal theory of laser refractive surgery is that the optical power of the eye can be changed by modifying the corneal curvature [1]. Laser in situ keratomileusis (LASIK) is performed to correct myopia and myopic astigmatism by ablating the corneal tissue and flattening the central anterior corneal curvature. The subsequent increase in the corneal radius lowers the dioptric power of the cornea and allows accurate correction of myopic defects [2].

Although laser in situ keratomileusis (LASIK) has been shown to be safe and effective for the treatment of myopia [3, 4], greater outcome variability has been reported in eyes with higher degrees of myopia [5].

Many factors have been investigated to find what influences the predictability of LASIK including patient age [6], optical zone diameter [6], epithelial hyperplasia [7], and preoperative keratometry (*K*) [5].

The question of whether the preoperative *K* power influences outcomes in myopic patients has been studied somewhat in patients undergoing photorefractive keratometry (PRK) procedures and more in hyperopic LASIK [8], and the findings are contradictory. Therefore, we tried to deduct the effect of both pre- and postoperative keratometry on moderate to high myopic patients undergoing LASIK since the literature is unclear regarding their influence.

## 2. Patients and Methods

The case records of 812 consecutive eyes of 420 patients with moderate and high myopia ≥−6 D were retrospectively analyzed. LASIK was done at Sohag Laser Center, Egypt, from January 2010 to November 2013. Data obtained from the case records included patient age, spherical equivalent (SE) refraction (pre- and postoperative), corrected distance visual acuity (CDVA) (pre- and postoperative), and keratometry (pre- and postoperative) and details of intraoperative complications. Follow-up was repeated at 3 and 6 months postoperatively. Exclusion criteria were (1) eyes with spherical equivalence >−12 D, (2) eyes with follow-up of less than 6 months, (3) eyes with intraoperative complications, (4) eyes with preoperative CDVA <0.3, (5) eyes that needed undercorrection due to thin corneas not permitting total correction, and (6) eyes with previous ocular surgeries. Rabinowitz criteria [9–11] were applied meticulously to screen for keratoconus and exclude risk factors.

Corneal keratometry (*K*) was measured in the flat and steep axes using a Scheimpflug topography system (Sirius, CSO, Italy). Pre- and postoperative average *K* = (*K* flat + *K* steep)/2. Change in *K* (Δ*K*) was calculated as preoperative minus postoperative average *K*. Visual acuity was recorded using logMAR.

## 3. LASIK Procedure

One refractive eye surgeon (EM) operated on the patients using the same nomogram for all treatments. The minimum residual stromal bed was planned to be 280 *μ*m with emmetropia being the goal in all cases. Superior hinged lamellar flaps were created with a Moria M2 microkeratome using a 90 *μ*m single use head and 9.0 mm ring. Laser ablation was performed using the VISX Star S4 IR, creating a 6.5 mm optical zone with 8.0 mm blend zone with centration over the center of the pupil. Following noncustomized ablation, the flap was replaced and the patients then received fluoroquinolone QID for 7 days and prednisolone acetate 1% drops QID for 7 days and then tapering over 3 weeks. Our final outcomes were compared at the 6-month follow-up.

For the purpose of analysis, patients were divided into three groups based on the degree of preoperative myopia: Group 1, −6 D to −7.9 D; Group 2, −8 to −9.9 D; and Group 3, −10 to −12 D. The reported refractive values correspond to spectacle plane. The refractive outcome among the different myopic groups was further stratified by pre- and postoperative keratometry. The study was approved by the ethical committee of Sohag Faculty of Medicine.

Statistical analysis was performed using the SPSS program. Means were compared using the unpaired *t*-test (2-tailed), while nonparametric data were analyzed using the chi-square test. Trends in data were tested using an analysis of variance, and the relationship between preoperative keratometry and postoperative refraction was studied by linear regression.

## 4. Results

The demographics of the 420 patients (155 men, 265 women) and the three groups which are stratified according to the degree of preoperative myopia are reported in Table 1. No statistically significant difference existed between the 3 myopic matched cohort groups in mean age (*P* = 0.26).

### 4.1. Preoperative Keratometry

**Figure 1:**
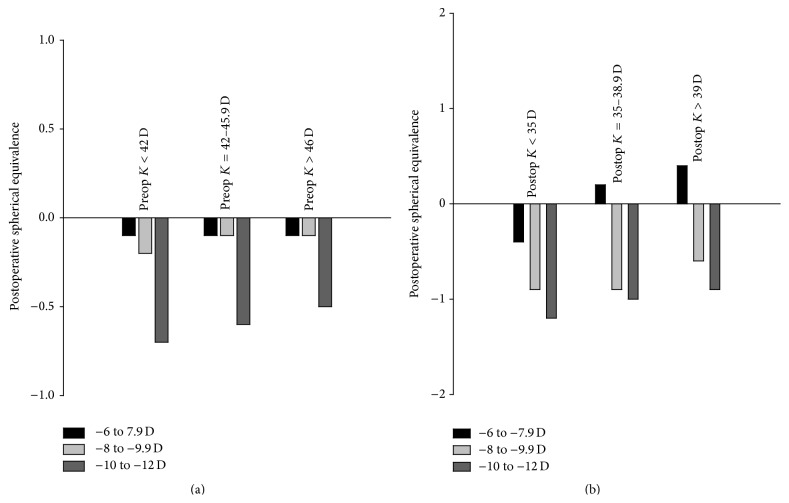
Postoperative spherical equivalence stratified by pre- and postoperative keratometry, respectively.

The data was further stratified according to the mean preoperative keratometry (*K*) power into 3 subdivisions: (1) *K* < 42 D, (2) *K* = 42–45.9 D, and (3) *K* > 46 D. There was a statistically significant difference in postoperative spherical equivalence in both groups, 2 and 3, when the corneal power was less than 42 D and more than 46 D (*P* = 0.032 and *P* = 0.015, resp.) (Figure 1(a)) and the same applies to the corrected distance visual acuity (Table 2). In patients with similar preoperative myopia, greater undercorrection was noted in eyes with preoperative keratometry <42 D. Although this was seen in all myopia subgroups, the difference was greatest in those with higher preoperative myopia (SE of –10.0 to –12 D) (*P* = 0.015).

### 4.2. Postoperative Keratometry

In Table 3, the postoperative corneal powers were divided into three subdivisions: (1) *K* < 35 D, (2) *K* = 35–38.9 D, and (3) *K* > 39 D. There was a statistically significant difference in postoperative spherical equivalence and corrected distance visual acuity in all groups of myopia when the corneal power was less than 35 D and more than 38 D. In patients with similar preoperative myopia, greater undercorrection was noted in eyes with postoperative keratometry <35 D than in eyes with steeper corneas (keratometry > 39 D) (Figure 1(b)).

### 4.3. Change in Keratometry (Δ*K*)

The impact of change in corneal power was also addressed. The results showed that the larger the change in the keratometry between pre- and postoperative keratometric readings is, the more the postoperative spherical equivalence was affected. The postoperative SE was more when Δ*K* was higher in all groups of myopia as shown in Table 4.

### 4.4. Corneal Asphericity

**Figure 2:**
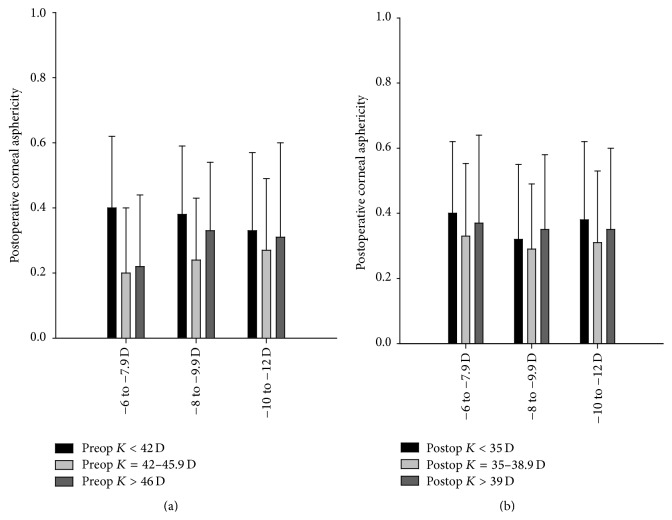
Postoperative corneal asphericity stratified by pre- and postoperative keratometry, respectively.

The results of Spearman correlation coefficient between the degree of pre- and postoperative keratometry and the *Q*-factor (pre- and postoperative) revealed a correlation coefficient of 0.90 and 0.96, respectively, indicating a high correlation between the 2 variables; that is, the change in corneal asphericity increased with the degree of corneal curvature (Figure 2).

## 5. Discussion

The precision of refractive surgery for the correction of myopia has improved with the advent of LASIK, particularly for high myopia. However, marked variability in refractive outcomes still exists among individual patients [3, 5].

This study analyzed the relationship between preoperative and postoperative keratometry to postoperative spherical equivalence and corrected distance visual acuity in a cohort of 812 eyes having LASIK by a single surgeon, using a standardized treatment protocol for all patients. To the best of our knowledge, this is the first study to address the effect of both pre- and postoperative keratometry in cases of moderate and high myopia in a large group of patients. We also addressed the effect of preoperative and postoperative corneal power on corneal asphericity.

The relationship between preoperative keratometry (*K*) and visual outcomes in laser-assisted in situ keratomileusis (LASIK) has been studied in myopia as well as in hyperopia.

We noted a trend toward greater undercorrection in patients with keratometry <43.5 D than in those with keratometry >46 D in all myopia groups. The possibility of undercorrection resulting from treating flatter corneas with a standard protocol has been described in a neural network model [12]. Also, the loss of ablation efficiency at nonnormal incidence may explain many of the current findings: Considering the loss of efficiency in a pure myopia profile, the profile “shrinks,” steepening the average slope and then slightly increasing the myopic power of the profile as well as inducing spherical aberrations [13–16].

Rao et al. [17] reported increased undercorrection in eyes with preoperative SE of −10.0 to −11.9 D and in eyes with flat corneas compared with steeper corneas. Perez-Santonja et al. [5] also reported a tendency toward undercorrection in eyes with flatter corneas that had received LASIK for the correction of high myopia of −8.00 to −20.00 D, while Christiansen et al. [18] studied moderately myopic eyes undergoing LASIK and their results suggested that flatter corneas have better visual outcomes than those with steeper corneas which disagreed with the previous studies as well as ours.

Other studies examining hyperopic LASIK agree with our results. Williams et al. [8] prospectively examined 6-month follow-up data and found an increased incidence of loss of BSCVA with eyes that had preoperative *K* > 44.0 D. Esquenazi and Mendoza [19] found that undercorrection occurred more frequently in eyes with preoperative *K* > 45.0 D.

Another study of the effect of keratometry on refractive outcome was carried out by de Benito-Llopis et al. [20] in LASEK cases on 1180 eyes and found that there is a weak positive correlation between preoperative keratometry and postoperative SE, mostly in the subgroup with steeper corneas and when the preoperative refractive error was higher. Yet they did not find a tendency toward undercorrection in flatter corneas, as some studies of LASIK have concluded. Studies by Hersh et al. [21], Blaker and Hersh [22], and Varssano et al. [23] yielded the same results in PRK patients as well. In contrast, Ditzen et al. [24] reported that preoperative *K* values affected outcomes of hyperopic LASIK. They found that flat, rather than steep, preoperative corneal keratometry led to greater regression and vision undercorrection.

The differences between the results of surface ablation and LASIK may be due to the fact that creating a stromal flap could be a confounding factor in LASIK. Flattening of the central cornea can result from cutting the peripheral stroma due to interlamellar forces [25, 26]. In non-femto-assisted LASIK, the microkeratome produces a meniscus shaped flap that is thinner in the center and thicker in the periphery [27, 28]. The preoperative corneal curvature seems to affect the profile of the stromal flap, while surface laser ablation alters only the corneal curvature, thus avoiding this potential confounding factor.

Another factor that might explain why the studies of PRK patients found no relationship between these 2 factors is the aggressive stromal healing response and epithelial remodeling in surface ablation. So, with less healing response in LASIK, any relationship between preoperative keratometry and final refraction may be more evident [29].

Rao et al. [17] suggest that because the change in corneal curvature is responsible for correcting myopia, more ablation might be required in a flatter cornea than a steeper cornea to produce a similar amount of effective change. Stark et al. [30] also suggest that the laser beam incises less perpendicularly the surface in the midperiphery of steep corneas than of flatter ones, which may cause a loss of ablative efficiency away from the corneal apex in steep corneas. This can result in deeper central ablation and shallower paracentral ablation leading to slightly greater myopic correction in steep corneas than in flat corneas.

As regards the effect of postoperative keratometry on refractive state, Jin et al. [31] found no association between postoperative *K* greater than 49.0 D and poor visual acuity in hyperopes. Cobo-Soriano et al. [32] also found that postoperative *K* did not affect outcomes and that postoperative *K* greater than 48.0 D had no effect on visual outcomes.

Tabbara et al. [33] supported the conclusion that rather than dependence on the preoperative or postoperative *K* values, the outcomes of hyperopic LASIK are dependent on intraoperative changes in *K*. Cobo-Soriano et al. as well found an association between *K* changes greater than 4.0 D and poor outcomes.

As regards corneal asphericity, Holladay et al. [34] found a high average rise in the *Q*-factor after refractive surgery, which declined over 6 months to a value that was still higher than the presurgery level.

Bottos et al. [35] investigated the asphericity change after wavefront-guided LASIK in 177 myopic eyes and found that there is a change in the direction of a more oblate profile which is in agreement with our results as well. Several studies revealed that corneal vertex centration resulted in less ocular aberrations and changes in asphericity. Because of the smaller angle between corneal vertex and pupil center associated with myopes compared with hyperopes, centration problems are less apparent [36, 37]. However, pupillary offset larger than 250 *μ*m seems to be sufficiently large to be responsible for differences in ocular aberrations, yet not large enough to correlate this difference in ocular aberrations with functional vision [38–40].

We classified the myopic eyes into 3 groups to avoid masking the underlying relationship between keratometry and refractive outcome if it has been analyzed all in one group as the level of preoperative ametropia was proved to strongly affect the final outcome [3, 5, 17]. We understand that longer follow-up periods were warranted to shed light on the long term postoperative effects.

In conclusion, our data revealed the occurrence of greater undercorrection after LASIK in eyes with flatter preoperative keratometry and postoperative keratometry as well. Analysis of large group of patients allowed us to conclude that the effect of preoperative keratometry on the final refractive outcome appeared greater in eyes with higher myopia, and these differences were clinically significant in eyes with myopia greater than –10.0 D. Preoperative and postoperative keratometry appeared to influence the corneal asphericity as well. LASIK nomograms integrating corneal curvature would lead to better outcomes particularly in eyes with high myopia.

## Figures and Tables

**Table 1:** Demographics of all eyes and the three groups stratified by degree of myopia.

Myopia group (D)	Patient age (years) (mean ± SD)	Number of eyes	Preoperative SE (D) (mean ± SD)	Preoperative keratometry (D) (Mean ± SD)	Postoperative SE (mean ± SD)	Postoperative keratometry (D) (mean ± SD)
All eyes	21.8 ± 5.2	812	−8.5 ± 3.2	42.3 ± 4.1	−0.13 ± 1.1	36.36 ± 3.8
−6 to −7.9 D	22.8 ± 8.1	370	−7.2 ± 0.4	44.2 ± 1.7	0 ± 1.1	36.9 ± 4.5
−8 to −9.9 D	21.1 ± 6.9	289	−9.1 ± 0.2	45.2 ± 1.9	−0.1 ± 1.4	36.2 ± 2
−10 to −12 D	21.5 ± 7.2	153	−11.00 ± 0.5	44.9 ± 1.4	−0.3 ± 1.00	36 ± 3.3

SE: spherical equivalence; D: Diopter.

**Table 2:** Postoperative CDVA stratified by preoperative keratometry.

Myopia group (D)	Postoperative CDVA (log MAR)			*t*-test comparing eyes with Preop *K* <42 D and >46 D	ANOVA comparing 3 groups with different Preop *K*
	Mean ± SD				
	Preop *K* <42 D	Preop *K* = 42 to 45.9 D	Preop *K* >46 D		
	(number of eyes)	(number of eyes)	(number of eyes)		
−6 to −7.9 D	0.0 ± 0.11 (109)	0.0 ± 0.12 (119)	0.0 ± 0.16 (142)	0.21	0.657
−8 to −9.9 D	0.05 ± 0.15 (66)	0.0 ± 0.13 (114)	0.11 ± 0.2 (109)	0.032^*∗*^	0.27
−10 to −12 D	0.12 ± 0.29 (28)	0.18 ± 0.29 (58)	0.18 ± 0.4 (67)	0.015^*∗*^	0.04^*∗*^

CDVA: corrected distance visual acuity; Preop: preoperative; *K*: keratometry; ANOVA: analysis of variance.

**Table 3:** Postoperative CDVA stratified by postoperative keratometry.

Myopia group (D)	Postoperative CDVA			*t*-test comparing eyes with Postop *K* <35 D and >39 D	ANOVA comparing 3 groups with different Postop *K*
	Mean ± SD				
	Postop *K* <35 D	Postop *K* = 35–38.9 D	Postop *K* >39 D		
−6 to −7.9 D	0.0 ± 0.6	0.0 ± 0.6	0.0 ± 0.5	0.05^*∗*^	0.42
−8 to −9.9 D	0.1 ± 1.2	0.1 ± 1	0.0 ± 1.1	0.033^*∗*^	0.47
−10 to −12 D	0.13 ± 1.3	0.1 ± 1.2	0.1 ± 1.2	0.02^*∗*^	0.15

CDVA: corrected distance visual acuity; Postop: postoperative; *K*: keratometry; ANOVA: analysis of variance.

**Table 4:** Postoperative spherical equivalence stratified by change in keratometry.

Myopia group (D)	Postoperative SE (D)			*P* value
	Mean ± SD			
	Δ*K* < 5 D	Δ*K* = 5–7.9 D	Δ*K* > 8 D	
−6 to −7.9 D	−0.4 ± 0.5	+0.2 ± 0.6	Nil	0.05^*∗*^
−8 to −9.9 D	Nil	−0.9 ± 1.2	−0.6 ± 1.1	0.033^*∗*^
−10 to −12 D	Nil	−1.0 ± 1.2	−0.9 ± 1.2	0.02^*∗*^

Δ*K*: change in keratometry; SE: spherical equivalent; ANOVA: analysis of variance.
